# Reconstituting NK Cells After Allogeneic Stem Cell Transplantation Show Impaired Response to the Fungal Pathogen *Aspergillus fumigatus*

**DOI:** 10.3389/fimmu.2020.02117

**Published:** 2020-09-10

**Authors:** Esther Weiss, Jan Schlegel, Ulrich Terpitz, Michael Weber, Jörg Linde, Anna-Lena Schmitt, Kerstin Hünniger, Lothar Marischen, Florian Gamon, Joachim Bauer, Claudia Löffler, Oliver Kurzai, Charles Oliver Morton, Markus Sauer, Hermann Einsele, Juergen Loeffler

**Affiliations:** ^1^Department of Internal Medicine II, WÜ4i, University Hospital Wuerzburg, Würzburg, Germany; ^2^Department of Biotechnology and Biophysics, Biocenter, Julius-Maximilian-University, Würzburg, Germany; ^3^Leibniz Institute for Natural Product Research and Infection Biology – Hans Knoell Institute, Jena, Germany; ^4^Institute for Hygiene and Microbiology, Julius-Maximilian-University, Würzburg, Germany; ^5^School of Science, Western Sydney University, Campbelltown, NSW, Australia

**Keywords:** natural killer cell, *Aspergillus fumigatus*, stem cell transplantation, corticosteroids, CCL3, CCL4, CCL5

## Abstract

Delayed natural killer (NK) cell reconstitution after allogeneic stem cell transplantation (alloSCT) is associated with a higher risk of developing invasive aspergillosis. The interaction of NK cells with the human pathogen *Aspergillus (A.) fumigatus* is mediated by the fungal recognition receptor CD56, which is relocated to the fungal interface after contact. Blocking of CD56 signaling inhibits the fungal mediated chemokine secretion of MIP-1α, MIP-1β, and RANTES and reduces cell activation, indicating a functional role of CD56 in fungal recognition. We collected peripheral blood from recipients of an allograft at defined time points after alloSCT (day 60, 90, 120, 180). NK cells were isolated, directly challenged with live *A. fumigatus* germ tubes, and cell function was analyzed and compared to healthy age and gender-matched individuals. After alloSCT, NK cells displayed a higher percentage of CD56^bright^CD16^dim^ cells throughout the time of blood collection. However, CD56 binding and relocalization to the fungal contact side were decreased. We were able to correlate this deficiency to the administration of corticosteroid therapy that further negatively influenced the secretion of MIP-1α, MIP-1β, and RANTES. As a consequence, the treatment of healthy NK cells *ex vivo* with corticosteroids abrogated chemokine secretion measured by multiplex immunoassay. Furthermore, we analyzed NK cells regarding their actin cytoskeleton by Structured Illumination Microscopy (SIM) and flow cytometry and demonstrate an actin dysfunction of NK cells shown by reduced F-actin content after fungal co-cultivation early after alloSCT. This dysfunction remains until 180 days post-alloSCT, concluding that further actin-dependent cellular processes may be negatively influenced after alloSCT. To investigate the molecular pathomechansism, we compared CD56 receptor mobility on the plasma membrane of healthy and alloSCT primary NK cells by single-molecule tracking. The results were very robust and reproducible between tested conditions which point to a different molecular mechanism and emphasize the importance of proper CD56 mobility.

## Introduction

Following allogeneic stem cell transplantation (alloSCT), patients are characterized by a period of profound T and B cell deficiency until the completion of immune system reconstitution, which can take up to 2 years (1). During this critical period of T and B cell deficiency, in which a patient is more susceptible to bacterial, viral and fungal infections, cellular components of innate immunity are of major importance as they represent the first line of immune defense. Alveolar macrophages can kill inhaled conidia, but additionally, recruit neutrophils to the site of infection. Phagocytes, such as monocytes and dendritic cells, communicate with the adaptive immune system, thereby modulating inflammatory responses. Through the involvement of pattern recognition receptors that recognize pathogen-associated molecular patterns, cells of the innate immune system distinguish between different pathogens (2–4). Cytokines, chemokines, and chemokine receptors play essential roles in immunity against opportunistic pathogens and may determine the type of effector response. NK cells develop in the bone marrow and secondary lymphoid organs until they reach a defined stage, enter the bloodstream, and seed into peripheral organs (5).

Interestingly, NK cells are not restricted after migration but can circulate from lung to blood and *vice versa*, as shown in the study by Marquardt et al. (6). NK cells constitute approximately 10% of lymphocytes in blood (7) and can be subdivided into CD56^bright^ and CD56^dim^ cells (8). CD56^bright^ NK cells are potent cytokine producers and do not express the Fc receptor CD16, while the latter efficiently lyse target cells (8). There is strong evidence for a gradual differentiation from CD56^bright^ cells into CD56^dim^ NK cells (9–11). Under healthy conditions CD56^dim^ NK cells represent ~90% of blood NK cells (7). In allograft recipients CD56^dim^ NK cells are underrepresented compared to healthy age and gender-matched controls as the subset ratio of CD56^bright^ NK cells increase to 40–50% (12). NK cells are potent killers of tumor cells, and once they encounter these cells, NK cells are activated and release the effector molecules granzyme and perforin, leading to target cell lysis (13). In addition to their tumor-killing function, NK cells also interact with several pathogens (14–16).

NK cells are essential during fungal infections, which has been shown in mouse models as well as in clinical studies. Morrison et al. showed in a neutropenic mouse model that CCL2 depletion inhibited pulmonary NK cell migration and furthermore favored the development of invasive aspergillosis (17). In a later study, NK cell-derived IFN-γ was shown to be essential to control fungal infections in neutropenic mice and depletion of NK cells or IFN-γ resulted in a higher fungal burden (18). Confirming the importance of NK cells in humans, Stuehler et al. monitored recipients of an allograft over 12 months and found a clear correlation between reduced NK cell counts and delayed NK cell reconstitution with a higher risk of developing IA in patients receiving HSCT (19). These studies highlighted the role of NK cells during fungal infections in immunocompromised hosts and were influential for our study.

Fungal pathogens are recognized by several natural activating receptors, e.g., natural activating receptor NKp46 and NKp30 (16, 20). A recent study demonstrated that the neural cell adhesion molecule (NCAM-1, CD56) on NK cells binds to hyphae of *Aspergillus* species which induces NK cell activation measured by the expression of the activation marker CD69 (21). Interestingly, blocking of CD56 resulted in reduced secretion of the chemokines macrophage inflammatory protein (MIP)-1α (CCL3), MIP-1β (CCL4), and RANTES (CCL5) after fungal challenge, suggesting inhibited immune cell recruitment to sites of inflammation (21).

In this study, we describe the process of NK cell reconstitution in randomly selected recipients of an allograft and present prospective longitudinal functional data from NK cells collected at defined time points after transplantation. We show that the expression of the fungal recognition receptor CD56 is increased for more than 180 days after alloSCT. Despite the higher expression, fungal binding was inhibited in some NK cells obtained from patients after alloSCT. We determined that this was not due to an actin defect; however, fungal mediated actin induction was dependent on time after alloSCT, indicating NK cell development-related effects. In additional experiments, we showed that corticosteroid treatment reduced the binding of CD56 to fungal pathogens and consequently diminished downstream chemokine secretion. By treatment of healthy, age and gender-matched NK cells with corticosteroids *ex vivo*, we confirmed that corticosteroids negatively influence CD56 downstream signaling by inhibition of chemokine secretion. Thus, corticosteroids may influence the development of IA by suppressing NK cell function in addition to effects on other immune cells. Interestingly, CD56 receptor mobility within the plasma membrane of alloSCT NK cells was not influenced as probed by single-molecule tracking experiments which indicates defects in CD56 signaling rather than its mobility.

## Materials and Methods

### Patient Information

Peripheral blood was collected from patients after the successful treatment of AML (acute myeloid leukemia), ALL (acute lymphatic leukemia) or multiple myeloma (MM). Drawing of blood was performed at day 60, 90, 120, and 180 post-alloSCT. Relevant patient information is displayed in Table 1.

**Table 1:** Patient characteristics.

**ID**	**Gender**	**Age (yrs)**	**Day 60**	**Day 90**	**Day 120**	**Day 180**	**GvHD with systemic CS treatment**	**CS treatment duration**	**CS dosage**
2	Female	36	CSA	CSA	CSA, pred	CSA, pred	No	>18 weeks	Low–dose
4	Male	59	CSA, MMF	CSA, MMF	CSA	noT	No	–	–
5	Male	62	MMF	pred	pred	noT	Skin	~4 weeks	Initial high doses
6	Female	60	CSA, MMF	CSA, MMF	CSA, hydrocort	CSA, hydrocort	No	>20 weeks	Low-dose
7	Male	57	MMF	noT	noT	noT	No	–	–
8	Female	49	CSA, MMF	CSA	CSA, Rituximab	CSA, pred, rapa	Skin, IT	~3 weeks	Initial high doses
9	Male	55	rapa, MMF, hydrocort, CSA	MMF, CSA	CSA	CSA, pred, rapa	Skin, IT	~4 weeks	Initial high doses
10	Male	63	pred, rapa	pred, rapa	pred, rapa	noT	No	>8 weeks	Low-dose
12	Female	45	CSA, MMF	n/a	n/a	n/a	No	–	–
14	Female	42	CSA, rapa, MMF	rapa, pred	rapa, pred	rapa, pred	Skin	>15 weeks	Initial high doses
15	Male	66	rapa, MMF, pred	rapa, MMF, pred	rapa, MMF, pred	rapa, pred	No	>25 weeks	Low-dose
16	Female	34	CSA, MMF	CSA, MMF	CSA, MMF, pred	Hydrocort	SKIN	~6 weeks	Initial high doses
17	Male	58	MMF, CSA, rapa	rapa, MMF	rapa	rapa	No	–	–
18	Male	45	CSA, pred, budesonid	rapa, pred	rapa, pred	n/a	liver, IT	>14 weeks	Initial high doses
22	Male	48	rapa, MMF	rapa, MMF	rapa, MMF	noT	No	–	–
24	Male	49	CSA, rapa	CSA, rapa	n/a	n/a	No	–	–
26	Male	65	CSA, MMF	CSA, MMF	CSA	CSA	No	–	–

*CSA, cyclosporine; rapa, rapamycin; hydrocort, hydrocortisone; pred, prednisolone; MMF, mycophenolate mofetil; CS, corticosteroid; IT, intestinal tract, low-dose (<1 mg/kg), high-dose (>1 mg/kg)*.

### Cell Culture

NK cells were isolated from EDTA blood obtained from healthy individuals or patients after 60, 90, 120, or 180 days post alloSCT. NK cell isolation was performed using the MACSxpress® Whole Blood NK Cell Isolation Kit (negative isolation, Miltenyi Biotec). NK cells were frozen in liquid nitrogen. Thawing of NK cells was performed in pre-warmed RPMI 1640 + 10% fetal calf serum (FCS). Cell recovery was over 90 % after thawing compared to the cell amount before freezing. NK cell viability after thawing was monitored with a cell viability analyzer (VICELL XR, Beckman Coulter) and was consistently over 95%. NK cells were pre-stimulated with 1,000 U/ml interleukin-2 (IL-2, Proleukin™ Novartis) overnight at a cell concentration of 1 × 10^6^ cells/ml. NK cells were cultured with *A. fumigatus* germ tubes (MOI 0.5) or plain medium (RPMI + 10 % FCS) at a cell concentration of 1 × 10^6^ cells/ml for 6 h. Cell cultures were harvested, centrifuged (300 g, 10 min), and supernatants were frozen at −20°C for short-term storage (22) for later enzyme-linked-immunosorbent immunoassay.

### Fungal Strain

The *A. fumigatus* strain ATCC46645 was plated on malt agar plates. Conidia were harvested and incubated in RPMI 1640 overnight under constant shaking (200 rpm) at 25 °C to generate *A. fumigatus* germ tubes. Germ tubes were centrifuged (5,000 g, 10 min) and resuspended in fresh medium supplemented with 10 % FCS.

### Flow Cytometry

NK cells were treated with the following antibodies to analyze the surface expression: anti-CD56 FITC (BD), anti-NKp46 PE (BD), anti-CD3 PerCP (BD), and anti-CD16 PerCP (Biolegend). To analyze the intracellular expression of phosphorylated NF-κB p65 peptide, NK cells were stained with surface antibodies, fixed and permeabilized according to the BD Cytofix/Cytoperm™ protocol, and were stained with PE mouse anti-NF-κB p65 (BD) antibody for 30 min. NK cell purity was monitored by NKp46^+^ and CD3^−^ gating and was consistently over 95% (21, 22). For analysis of actin dynamics in live cells, cells were stained in 1 μM Live Cell Fluorogenic F-actin Labeling Probe (SiR-actin 647, Spirochrome) for 50 min. Relative CD56 and F-actin values were calculated with equations (1) and (2). Flow cytometric analysis was performed with a FACSCalibur (BD), and data were analyzed by FlowJo software (TreeStar).

(1) $$\begin{array}{l} \text{Relative CD}56\;=\frac{\text{CD}56\text{ MFI }(\text{NKAF})}{\text{CD}56\text{ MFI }(\text{NK})} \end{array}$$

(2) $$\begin{array}{l} \text{Relative F}-\text{actin }=\frac{\text{SiR}-647\text{ MFI }(\text{NKAF})}{\text{SiR}-647\text{ MFI }(\text{NK})} \end{array}$$

### CD16^+^ Cell Isolation

NK cell subsets were separated by CD16 magnetic beads over an MS column (Miltenyi Biotec). CD16^+^ and CD16^−^ cells were pre-stimulated with 1,000 U/ml Proleukin overnight before co-cultures were set with *A. fumigatus* germ tubes for 6 h at 37°C. Supernatants were frozen at −20°C as previously described (22) and were used for later enzyme-linked immunosorbent assay. NK cells were analyzed by flow cytometry, and subset purity was monitored by anti-CD56 antibody staining to discriminate between the CD56^dim^ and the CD56^bright^ subset.

### Multiplex Immunoassay

Supernatants of NK cells from alloSCT patients and healthy age and gender-matched controls treated with *A. fumigatus* germ tubes or non-treated NK cells were analyzed by multiplex immunoassay (eBioscience) regarding the presence of the chemokines MIP-1α, MIP-1β, and RANTES using the BioPlex 200 System (Bio-Rad).

### Enzyme-Linked Immunosorbent Assay (ELISA)

Supernatants of healthy age and gender-matched NK cells treated with prednisolone *ex vivo* were analyzed regarding the presence of MIP-1α, MIP-1β, and RANTES. MIP-1α, MIP-1β (R&D Systems), and RANTES (Biolegend) ELISA were performed according to the manufacturer's protocol.

### Prednisolone Treatment

NK cells were incubated in 1,000 U/ml IL-2 in the presence of 6.26, 12.5, 18.75, or 25 μg/ml Prednisolut® (prednisolone 21-succinate, mibe GmbH) for 40 h. Prednisolut® concentrations were comparable to those administered to patients in an acute phase treatment (2 mg/kg). After prednisolone treatment, the medium was exchanged, and NK cells were either co-cultured with *A. fumigatus* germ tubes (MOI 0.5) or alone for 6 h. NK cells were analyzed by flow cytometry, and supernatants were used for ELISA.

### Structured Illumination Microscopy

NK cells were cultured alone or with *A. fumigatus* germ tubes (MOI 0.5) for 6 h at 37°C on poly-D-lysine coated 8-well Lab-Tek coverglass chambers (Sarstedt). Samples were fixed in 3% formaldehyde (FA) in RPMI 1640 before staining. CD56 was stained with anti-CD56 Alexa Fluor 647 antibody (1:50, clone HCD56, Biolegend) for 1 h, the actin cytoskeleton with Phalloidin Atto 550 staining solution for 24 h (1:100, Sigma), and the fungal cell wall with Calcofluor for 10 min (500 μg/ml, Sigma). Samples were embedded in ProLong Gold Antifade Mountant (Thermo Fisher Scientific) and analyzed on a Zeiss Elyra S.1 SIM. Using the Plan-Apochromat 63x/1.4 Oil objective in combination with 642 nm (5%), 561 nm (1%), and 405 nm (2%) laser lines, we acquired z-stacks with slice spacings of 300 nm. Exposure times of 100 ms and 3 SIM rotations were used for each individual channel. Emission signals were recorded with a sCMOS PCO Edge 5.5 and analyzed with the ZEN 2.3 SP1 software. For visualization maximum intensity projections of reconstructed z-stacks were used. To quantify the actin signal of each NK cell, z-stacks were projected as summed slices. A constant circular area was moved over each NK cell and the intensity was measured using Fiji (23).

### Single-Molecule Tracking

Single-molecule tracking was performed as described recently (24, 25). In short, CD56 primary antibodies (clone HCD56, Biolegend) were conjugated to the bright and photostable fluorophore SeTau-647-NHS (SETA BioMedicals, #K9-4149) (26) via N-hydroxysuccinimide ester amine crosslinking. Primary NK cells were labeled 5 min at 4°C with 0.33 nM antibody solution diluted in colorless RPMI 1640. Cells were washed via centrifugation at 500 × g and seeded onto KOH cleaned 8-well on cover glass (Sarstedt). After 30 min, single-molecule trajectories were recorded at room temperature by using a highly inclined and laminated optical sheet (HILO) and a previously described *d*STORM setup (27). To increase signal-to-noise ratio exposure times were set to 50 ms and time series with 5,000 frames acquired. Individual molecules were detected by ThunderSTORM (28), filtered and tracks generated with minimal track length of 20 frames with the Python implementation of the Crocker-Grier (29) algorithm Trackpy [(30) April 21]. Mean squared displacements were calculated and the resulting ensemble MSD was fit with a power law, MSD(τ) = ${4\text{D}}_{\text{α}}\tau^{\text{α}}$ resulting in the generalized diffusion coefficient D_α_ and anomalous exponent α.

### Statistical Analysis

The normality of the data was tested with D'Agostino and Pearson normality test. Normally distributed data (*n* = 2 groups) were either analyzed with an unpaired *t*-test with Welch's correction or with a paired *t*-test. Data not following Gaussian's distribution were analyzed with a Mann-Whitney test. When multiple comparisons were performed, normally distributed data were analyzed by one-way ANOVA with correction for multiple testing by FDR method of Benjamini and Hochberg. Data not following Gaussian's distribution were analyzed with a Kruskal-Wallis test with FDR correction (no data matching) or with a Friedman test with FDR correction (matched data). Statistical analysis was performed with GraphPad Prism 7.

### Ethical Statement

This study obtained written consent by the ethics committee of the University of Wuerzburg (#34/15).

### Data Availability

All relevant data supporting the findings of the study are available in this article and its Supplementary Information files, or from the corresponding authors upon request, with restriction of data that would compromise patient confidentiality.

## Results

### Characterization of NK Cells From Recipients of an Allograft and Healthy Age and Gender-Matched Controls

**Figure 1:**
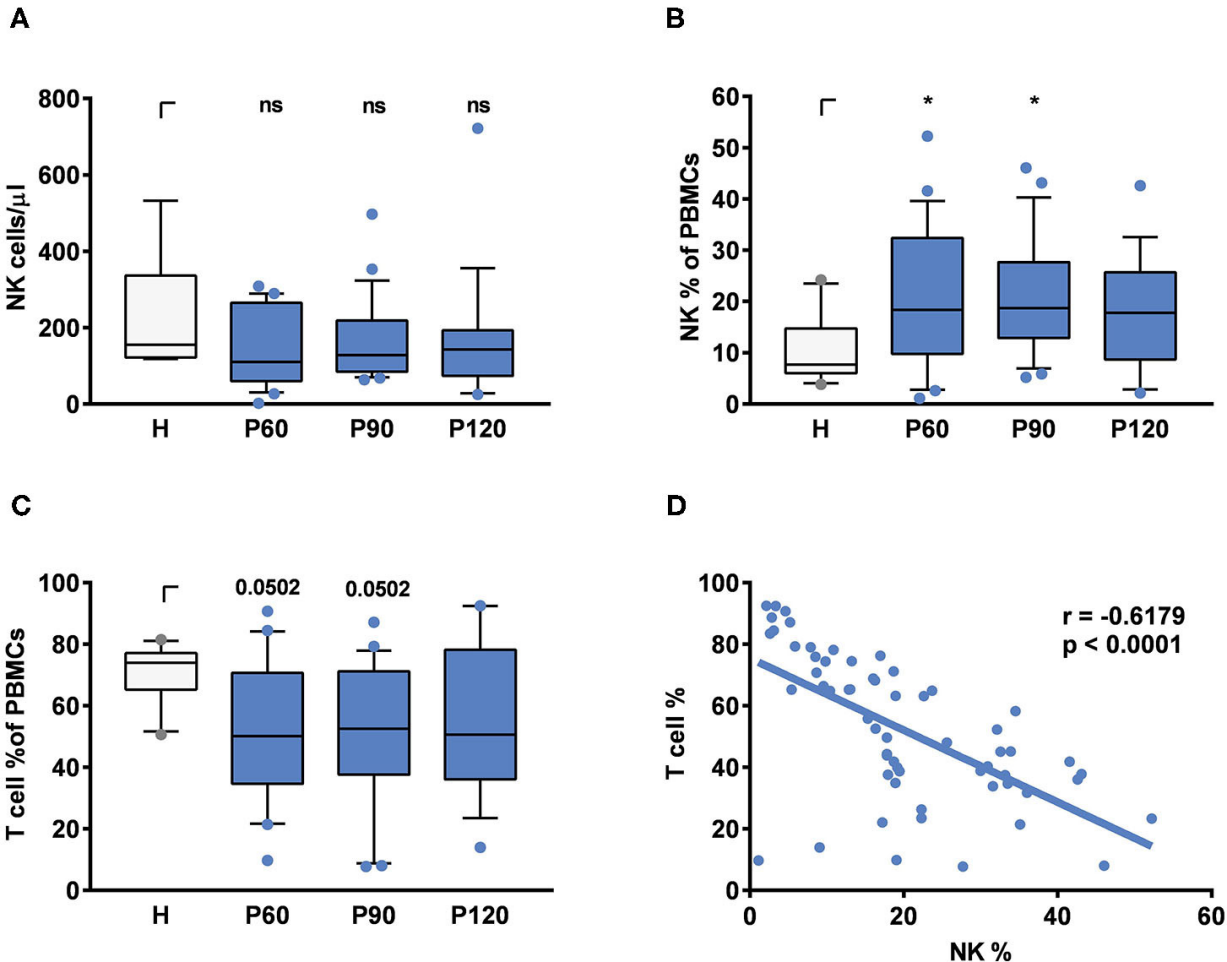
NK and T cell composition in patients after alloSCT and healthy controls. **(A)** Total NK cell counts per μl, **(B)** NK cell percentages in PBMCs and **(C)** T cell percentages in PBMCs were measured in the peripheral blood from healthy individuals (H) or patients 60, 90, or 120 days post-alloSCT (P60, P90, P120). Statistics were analyzed by **(A)** Kruskal-Wallis test with FDR correction (Benjamini and Hochberg), **(B,C)** one-way ANOVA with FDR correction [Benjamini and Hochberg, *q < 0.05, *F*_B_(3, 70) = 2.248, *F*_c(3,70)_ = 1.964]. Data were acquired from **(A–C)** *n* = 10 (H), *n* = 22 (P_60), *n* = 23 (P_90), and *n* = 19 (P_120) independent experiments. **(D)** NK/ T cell ratios were calculated by Pearson correlation. Data were obtained from *n* = 60 different experiments including time points ranging from 60 to 120 days post-alloSCT patients (*n* = 26).

To monitor NK cell counts and subsets in patients after successful treatment of AML (acute myeloid leukemia), ALL (acute lymphatic leukemia) or multiple myeloma (MM) by alloSCT and healthy donors, we analyzed blood at 60, 90, and 120 days after alloSCT and in healthy controls. In line with previous studies (31), total NK cell counts were similar within 60–120 days after transplantation compared to healthy controls (Figure 1A). Patients after alloSCT experience a period of immune deficiency in which T cells are underrepresented (1); thus, the percentage of other cell types in PBMCs increases. When the percentage of NK cells (Figure 1B) measured in PBMCs was plotted against the percentage of T cells after alloSCT (Figure 1C), a negative correlation was observed (p <0.0001), confirming that the higher percentage of NK cells in PBMCs was associated with T cell deficiency (Figure 1D).

**Figure 2:**
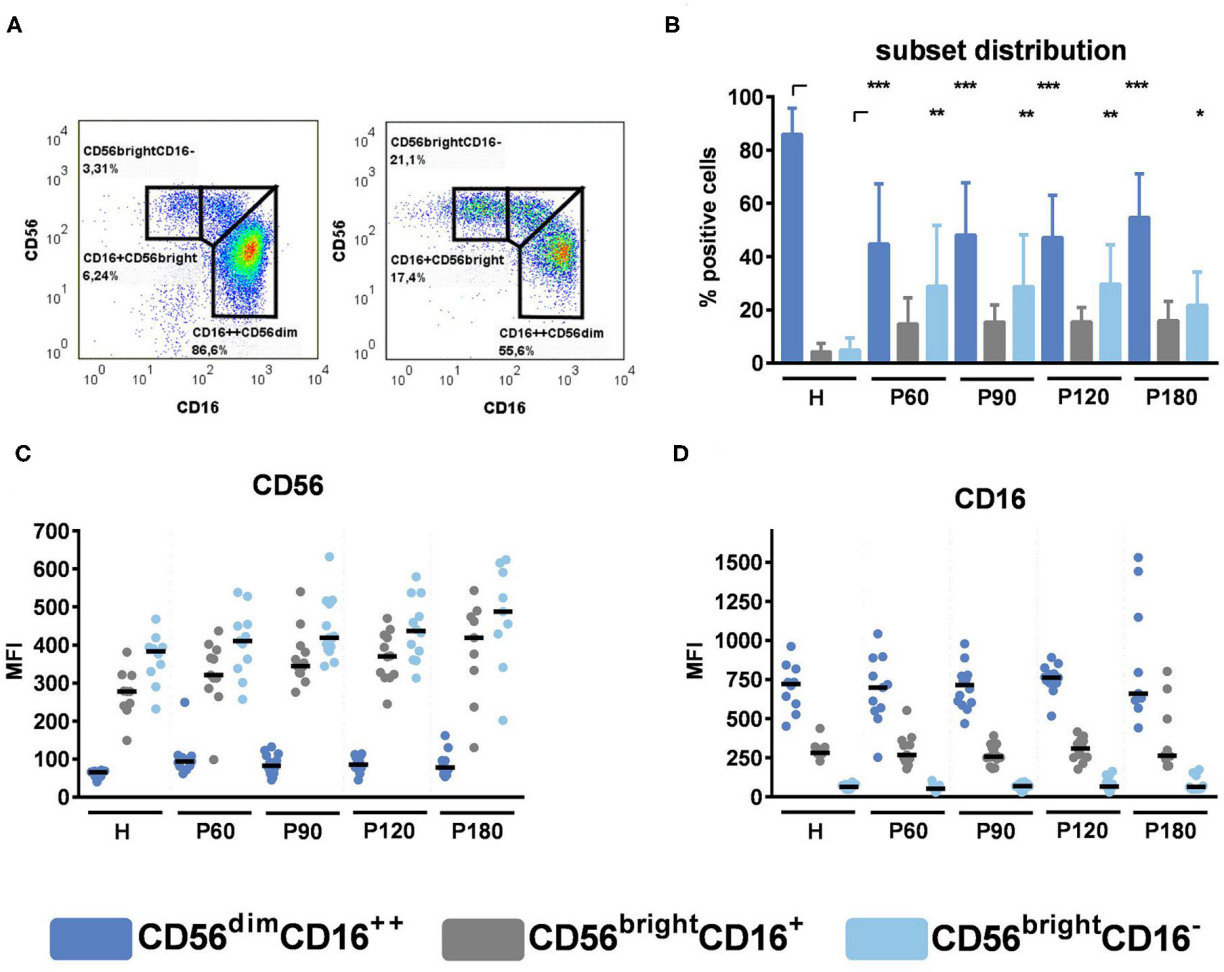
NK cell subsets in reconstituting NK cells. NK cells were pre-stimulated with 1,000 U/ml IL-2 overnight. **(A)** Representative gating strategies for healthy individuals (left) and recipients of an allograft (right). **(B)** NK cell subsets were compared between healthy individuals and recipients of an allograft. Data are displayed as mean + SD. **(C)** CD56 MFI and **(D)** CD16 MFI within NK cell subset were analyzed by flow cytometry. Data were acquired from *n* = 10 (H); *n* = 11 (P60); *n* = 14 (P90); *n* = 12 (P120); *n* = 9 (P180) different experiments. Medians are displayed. Statistical analysis was performed by **(B)** one-way ANOVA [CD16^++^CD56^dim^ and CD16^+^CD56^bright^, *F*_(14,156)_ = 25.87] and Kruskal-Wallis test (CD16^−^CD56^bright^). Corrections for multiple testing were performed by the FDR method of Benjamini and Hochberg. Statistical significances are marked by asterisks (*p < 0.05, **p < 0.01, ***p < 0.001).

NK cells obtained from healthy donors or after alloSCT were analyzed for the expression of specific surface markers (Figure 2). For this purpose, NK cells were stained with anti-CD56 and anti-CD16 antibodies and gated into CD56^bright^CD16^−^, CD56^bright^CD16^+^, and CD56^dim^CD16^++^ subsets (Figure 2A). NK cells derived after alloSCT significantly displayed lower amounts of CD56^dim^CD16^++^ cells, whereas the proportion of CD56^bright^CD16^+^ and CD56^bright^CD16^−^ cells was higher (Figure 2B), which is in accordance with previous findings (12). In contrast, the phenotypic expression of CD56 and CD16 within subsets remains unchanged after alloSCT, as there were no significant differences detectable when we analyzed the CD56 and CD16 MFI within each subset between healthy donors and recipients of an allograft (Figures 2C,D). These experiments showed that NK cell recovery after alloSCT is a fast process whereas NK cell subset distribution does not recover even by 180 days post-alloSCT.

### NK Cell Binding to the Fungus Is Inhibited by Corticosteroid Treatment

As the fraction of CD56^dim^ NK cells was lower in allograft recipients, we investigated the fungal binding capacity of reconstituting, not fully matured NK cells. CD56 is a PRR on human NK cells, and stimulation with *A. fumigatus* germ tubes causes relocalization of the normal homogenously distributed CD56 from the NK cell surface to the fungal interface, resulting in a reduced detection of CD56 by flow cytometry (21). This reduced detection of CD56 on NK cells that are derived from former NK-*A. fumigatus* co-culture experiments correlates with a remaining CD56 signal on the fungal surface in absence of NK cells (Supplementary Figure 1), concluding that CD56 stays stuck on the fungus after physical separation of co-cultures.

**Figure 3:**
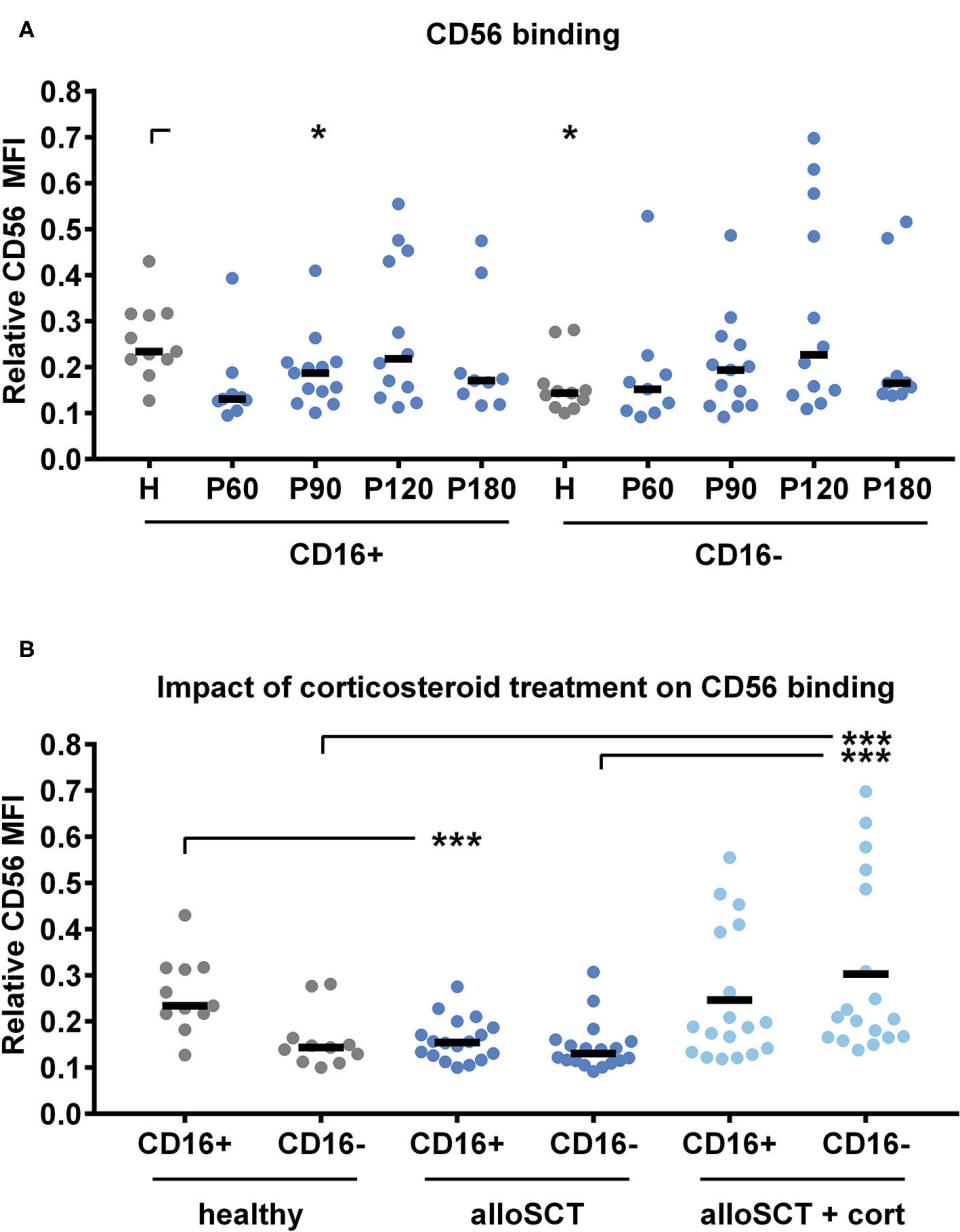
Corticosteroid treatment inhibits CD56 binding to the fungus. NK cells were pre-stimulated with 1,000 U/ml IL-2 overnight before NK cells were co-cultured with *A. fumigatus* germ tubes (AF, MOI 0.5) or plain medium at 37°C for 6 h. NK cells were gated into NKp46^+^/CD16^+^ or NKp46^+^/CD16^−^ cells. CD56 mean fluorescence intensity (MFI) was evaluated by flow cytometry with and without fungal co-culture. Relative CD56 expression was calculated for alloSCT NK cells (P) and healthy controls (H). Values for relative CD56 expression were grouped regarding **(A)** the time after alloSCT and **(B)** corticosteroid treatment. Data were acquired from **(A)** *n* = 11 (H); *n* = 9 (P60), *n* = 13 (P90); *n* = 12 (P120); *n* = 9 (P180) and **(B)** *n* = 11 (healthy) *n* = 18 (alloSCT and alloSCT + cort) experiments. Medians are displayed. Significant differences were calculated by the Kruskal-Wallis test with correction for multiple testing by the FDR method of Benjamini and Hochberg and are marked by asterisks (*p < 0.05; ***p < 0.001).

We investigated the fungal binding capacity by co-culturing NK cells from alloSCT recipients or healthy individuals with *A. fumigatus* germ tubes for 6 h before analyzing the cells by flow cytometry. To analyze the individual NK cell subsets we gated into CD16^−^ and CD16^+^ NK cells. Within CD16^−^ and CD16^+^ cells, we analyzed the fungal binding by CD56 mean fluorescence intensity (MFI). Relative CD56 values were calculated through dividing CD56 MFI after fungal co-culture by CD56 MFI before fungal co-culture. Thus, low relative CD56 values indicated a strong binding of CD56 (Figure 3).

Healthy CD16^−^ NK cells displayed lower relative CD56 values compared to healthy CD16^+^ NK cells, indicating better fungal binding of the CD16^−^ subset (Figure 3A). In contrast, there was no detectable difference between CD16^+^ and CD16^−^ cells in alloSCT patients (Figure 3A). The binding capacity of NK cells obtained after alloSCT did not significantly change over time; however, we detected outliers leading to high standard deviations in each group (Figure 3A). Next, we analyzed if those outliers correlate with a special treatment after alloSCT, as drugs may impair cell function. Within the CD16^+^ subset, more CD56^bright^ cells were observed in patients after alloSCT compared to healthy individuals (Figure 1B). This may be the reason why CD16^+^ cells after alloSCT (median = 0.15) displayed a better fungal binding compared to healthy CD16^+^ cells (median = 0.23) (Figure 3B). This enhanced fungal binding of CD16^+^ cells after alloSCT was abrogated when patients were under corticosteroid therapy during the time of blood collection (Figure 3B). In addition, a lower capacity in fungal binding was observed in the CD16^−^ subset derived from patients under corticosteroid therapy (CD16^−^ = 0.21) compared to non-corticosteroid treated individuals (CD16^−^ = 0.13) or even healthy individuals (CD16^−^ = 0.14) (Figure 3B).

### NK Cells Derived From Patients During Corticosteroid Therapy Display Reduced Fungal Mediated Chemokine Secretion

**Figure 4:**
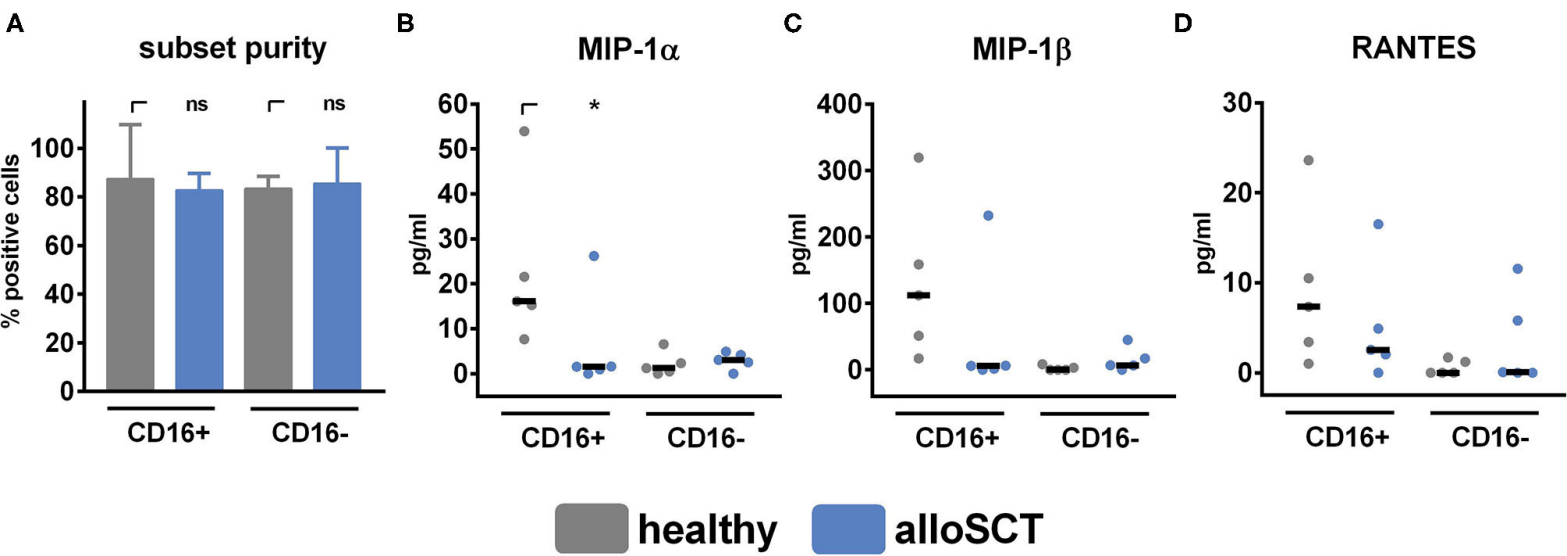
Chemokine secretion is inhibited in CD16^+^ cells after alloSCT. NK cells were obtained from healthy individuals or 90 days after alloSCT and were pre-stimulated with 1,000 U/ml IL-2 overnight. **(A)** NK cell subsets were isolated with CD16 magnetic bead positive isolation, and subset purity was analyzed by flow cytometry. NK cell subsets were co-cultured with *A. fumigatus* germ tubes (AF, MOI 0.5) or plain medium for 6 h at 37°C. Supernatants were collected, and chemokine levels of **(B)** MIP-1α, **(C)** MIP-1β, and **(D)** RANTES were analyzed. Data are displayed as **(A)** medians + range, **(B–D)** medians and were acquired from *n* = 5 different experiments. Significant differences were calculated by the Kruskal-Wallis test with correction for multiple testing by the FDR method of Benjamini and Hochberg and marked by an asterisk (*p < 0.05).

After CD56 binding to the fungus, NK cells secrete the chemokines MIP-1α, MIP-1β, and RANTES that are reduced secreted when CD56 is blocked before fungal co-culture (21). These chemokines are important for immune cell recruitment (32–34) and are secreted explicitly by CD16^+^CD56^dim^ NK cells, which are underrepresented early after alloSCT (Figure 2B) (35). To specifically analyze CD16^+^CD56^dim^ NK cells, we separated CD16^+^ from CD16^−^ cells by CD16 positive magnetic isolation (Figure 4A). CD16^+^ cells derived 90 days after alloSCT secreted significantly lower amounts of MIP-1α and showed a reduced secretion of MIP-1β and RANTES after fungal stimulation compared to healthy controls, indicating functional deficiencies of this subset (Figures 4B–D).

**Figure 5:**
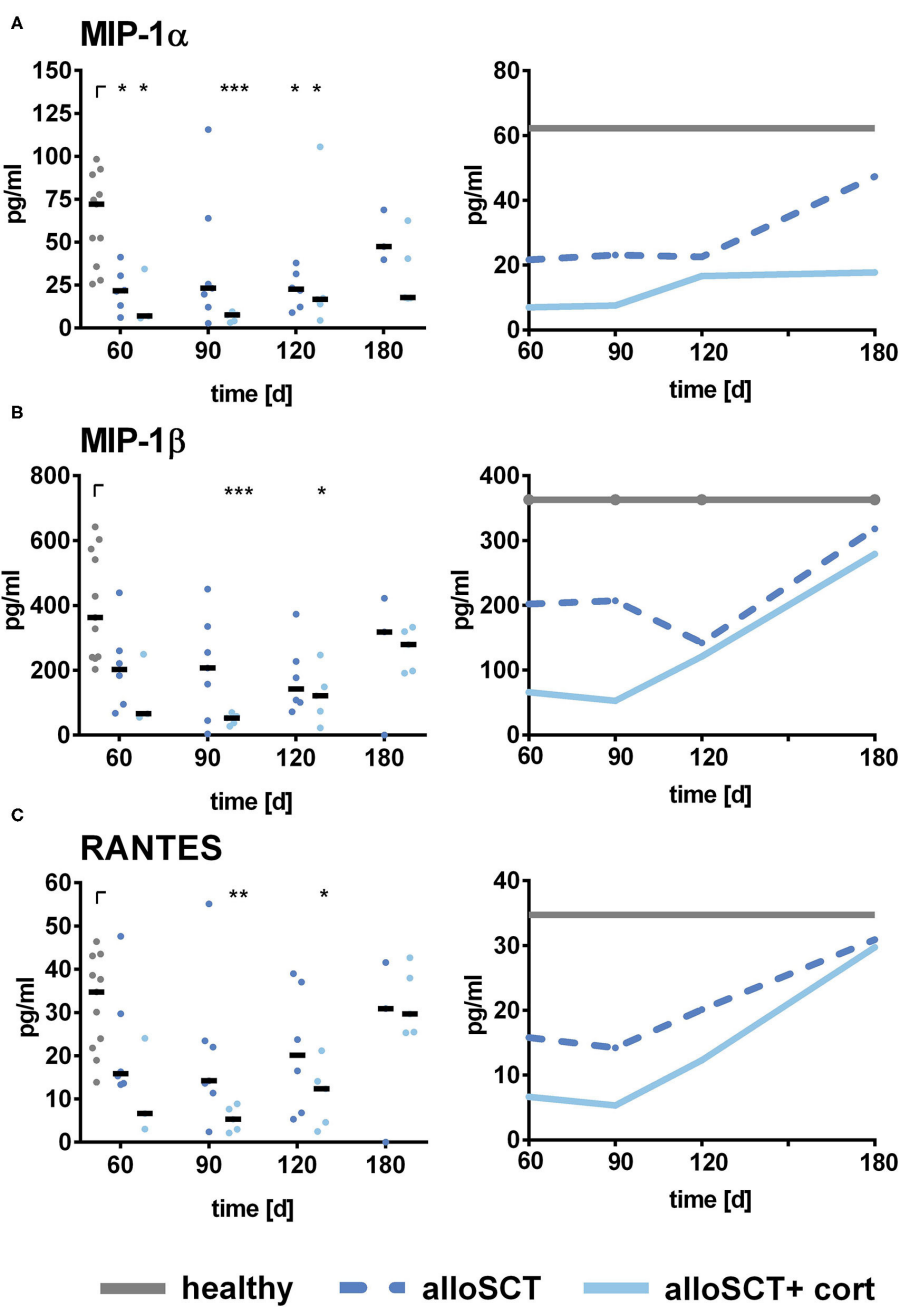
Chemokine secretion by alloSCT NK cells and healthy controls. NK cells were pre-stimulated with 1,000 U/ml IL-2 overnight. NK cells obtained after alloSCT with and without corticosteroid treatment (alloSCT + cort) or from healthy controls (H) were co-cultured with *A. fumigatus* germ tubes (AF, MOI 0.5) or plain medium at 37°C for 6 h. Supernatants were collected, and cytokine and chemokine levels of **(A)** MIP-1α, **(B)** MIP-1β, and **(C)** RANTES were analyzed. Data were acquired from *n* = 11 (H), *n* = 6 (alloSCT 60), *n* = 7 (alloSCT 90), *n* = 6 (alloSCT 120), *n* = 3 (alloSCT 180), *n* = 3 (cort 60), *n* = 5 (cort 90), *n* = 120 (cort 120), and *n* = 5 (cort 180) experiments. Data are displayed as medians. Significant differences were calculated by Kruskal-Wallis test with correction for multiple testing by the FDR method of Benjamini and Hochberg. Statistical significances are marked by asterisks (*p < 0.05, **p < 0.01, ***p < 0.001).

Since corticosteroids were negatively influencing CD56 binding to the fungus, which was shown to mediate the secretion of MIP-1α, MIP-1β, and RANTES (21), we analyzed if corticosteroids were also influencing chemokine secretion *in vivo*. Indeed, NK cells derived from patients after alloSCT that were currently under corticosteroid therapy secreted lower amounts of chemokines compared to NK cells derived from patients without corticosteroid therapy (Figure 5).

### NK Cells From Healthy Individuals Treated With Prednisolone *ex vivo* Show Impaired Fungal Mediated Chemokine Secretion

**Figure 6:**
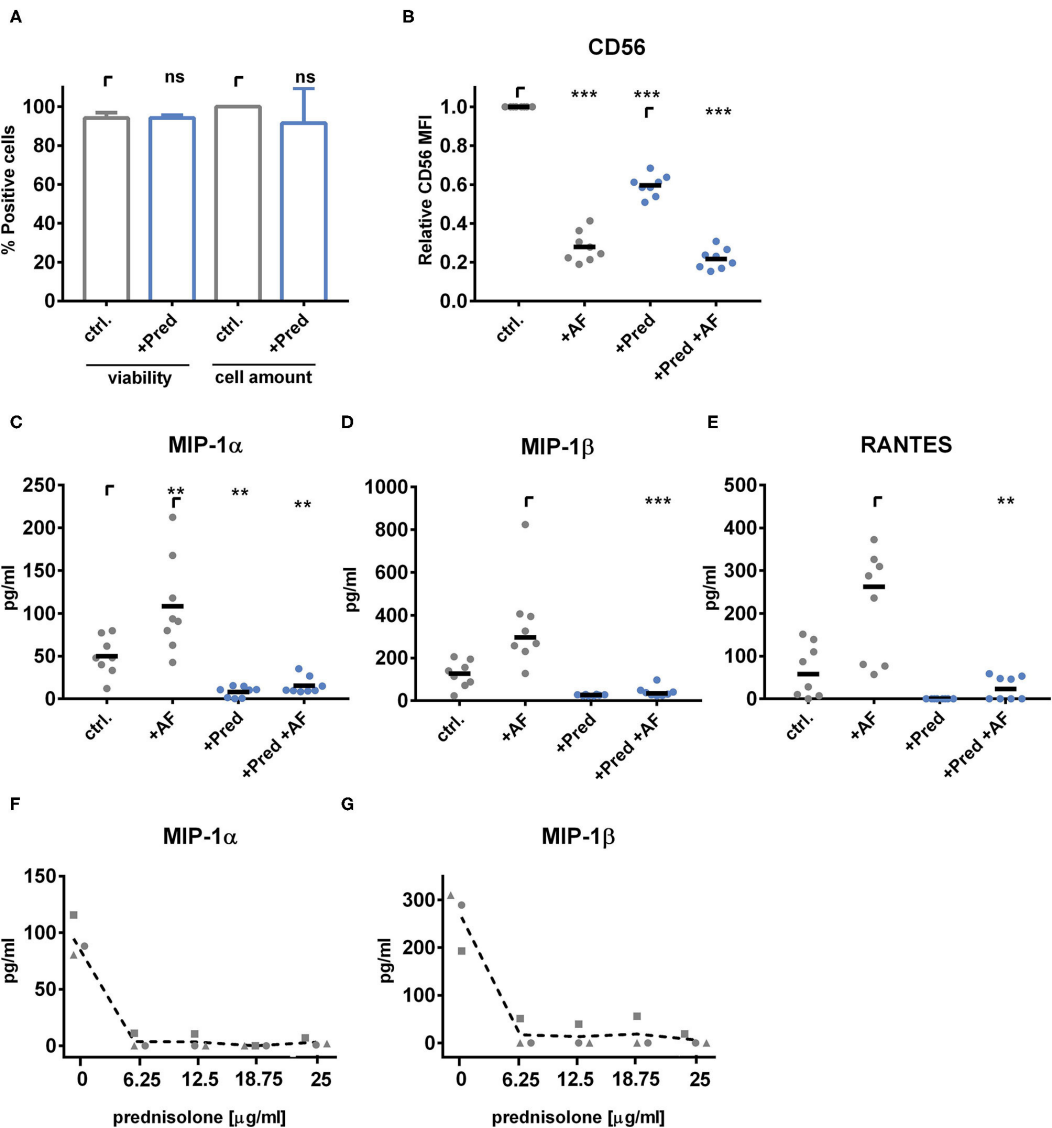
Prednisolone treatment of healthy NK cells reduces CD56 mediated chemokine secretion of MIP-1α, MIP-1β, and RANTES. NK cells were cultured with 1,000 U/ml IL-2 in the presence of 25 μg/ml prednisolone for 40 h. **(A)** NK cell viability and cell counts were monitored by trypan blue staining using a cell viability analyzer (Beckman Coulter Vicell XR). NK cell-*A. fumigatus* (MOI 0.5) co-cultures were set for 6 h. **(B)** CD56 mean fluorescence intensity (MFI) was analyzed by flow cytometry. The secretion of **(C)** MIP-1α and **(D)** MIP-1β and **(E)** RANTES was analyzed by multiplex immunoassay. Data were acquired from **(A)** *n* = 5, **(B–E)** *n* = 8 different experiments. Data are displayed as **(A)** means + SD, **(B,C)** means, and **(D,E)** medians. Statistics were calculated by **(A)** paired *t*-test, **(B,C)** one-way ANOVA with FDR correction [*F*_B(1.452,10.16)_ = 445.2, *F*_C(1.198,8.385)_ = 21.46] and **(D,E)** Friedman test with FDR correction. Statistical significances are marked by asterisks (**p < 0.01, ***p < 0.001). **(F,G)** NK cells from *n* = 3 healthy individuals were treated with increasing concentrations of prednisolone (0, 6.25, 12.5, 18.75, or 25 μg/ml) and were challenged with *A. fumigatus* germ tubes for 6 h. Supernatants were analyzed by ELISA for **(F)** MIP-1α and **(G)** MIP-1β.

Since we observed defects in CD56 binding and chemokine secretion by NK cells upon obtained from patients after corticosteroid treatment, we next analyzed the effects of corticosteroids on healthy NK cells *ex vivo*. Therefore, we stimulated NK cells for 40 h with levels of prednisolone corresponding to serum levels in patients during treatment of acute graft-vs.-host-disease. Prednisolone had no effect on either NK cell viability nor on the cell amount measured by a cell viability analyzer (Figure 6A). After stimulation, the medium was exchanged, and NK cells were co-cultured with *A. fumigatus* germ tubes for 6 h. Prednisolone induced the down-regulation of CD56 MFI on the surface of NK cells, which made relative CD56 MFI an unreliable readout for analyzing the *ex vivo* effect of prednisolone treatment on CD56 binding to the fungus (Figure 6B). Notably, NK cells derived from patients during corticosteroid therapy showed no CD56 downregulation compared to the control patient group (Supplementary Figure 2). A corticosteroid-mediated down-regulation of activation markers was shown in earlier studies (36). Besides CD56, the NK cell activation markers NKp46 and CD69 as well as intracellular phosphorylated NF-κB p65 peptide were down-regulated on the NK cell surface after prednisolone treatment *ex vivo* (Supplementary Figure 3).

Thus, we focused on the secretion of the fungal mediated chemokines MIP-1α, MIP-1β, and RANTES after treatment of healthy NK cells *ex vivo* with prednisolone. Interestingly, prednisolone treatment abrogated the ability to secrete these chemokines in fungal stimulated NK cells (Figures 6C–E). NK cells showed to be very sensitive to *ex vivo* corticosteroid treatment (Figures 6F,G). From these experiments, we concluded that corticosteroids have detrimental effects on fungal binding and secretion of chemokines.

### NK Cells Obtained After alloSCT Display Defects in Fungal Mediated Actin Polymerization

**Figure 7:**
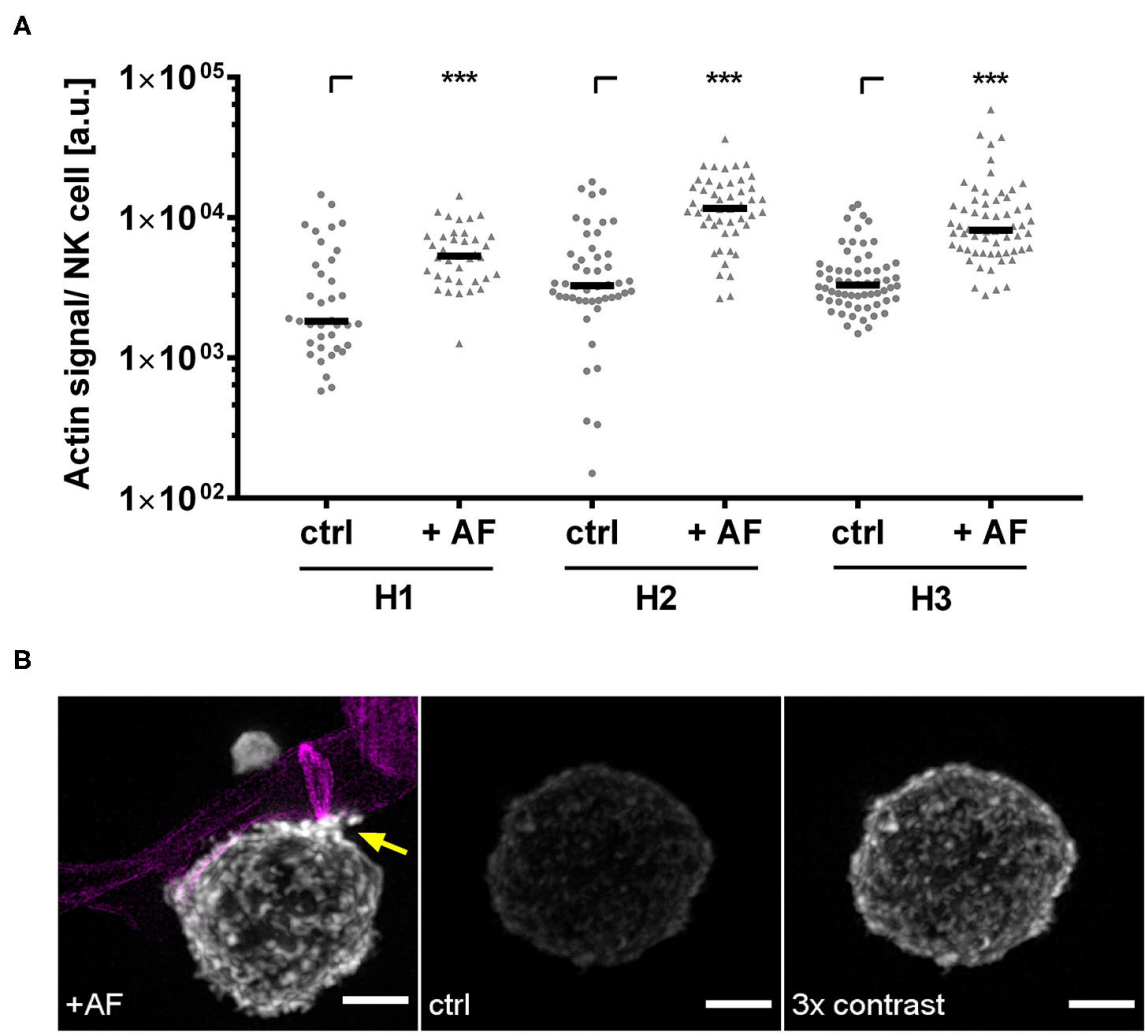
*A. fumigatus* stimulates F-Actin in NK cells. NK cells were pre-stimulated with 1,000 U/ml IL-2 overnight. NK cells were co-cultured with *A. fumigatus* germ tubes (AF, MOI 0.5) or without (ctrl) for 6 h. Cultures were fixed, and F-actin was stained with phalloidin staining solution for 24 h. Calcofluor was used to visualize the fungal cell wall. **(A)** Quantification of the actin signal per NK cell derived from *n* = 37 (H1), *n* = 48 (H2), and *n* = 62 (H3) SIM z-stacks. Data are displayed in medians and arbitrary units. Statistics were calculated by Wilcoxon test to compare the control samples and the fungal treated samples within each donor. Significant differences are displayed by asterisks (***p < 0.001). **(B)** Fluorescence intensities were compared between NK cells treated with *A. fumigatus* germ tubes (AF, left) or control cells (ctrl, middle). To better visualize the distribution of F-actin on control cells, we displayed the fluorescence signal of phalloidin with three times higher contrast (right). Increases in F-actin levels by fungal treatment are marked by an arrow. Fungal hyphae are displayed in magenta. Representative data from *n* = 3 different experiments are shown. Scale bar, 2 μm.

Our previous studies demonstrate that CD56 relocalization to the fungal interface is dependent on actin (21). Since NK cells obtained from patients treated with corticosteroids showed reduced CD56 relocalization (Figure 3), we hypothesized that this might be due to cytoskeletal defects. Thus, we first analyzed whether fungal stimulation induces actin polymerization by co-culturing NK cells obtained from healthy controls or after alloSCT with *A. fumigatus* germ tubes for 6 h. SIM was used to visualize actin dynamics in NK cells with subdiffraction spatial resolution (37). Indeed, in NK cells from healthy individuals the presence of *A. fumigatus* germ tubes induced actin polymerization as indicated by more intense fluorescent staining of the F-actin binding probe phalloidin (Figure 7A). In particular, the fluorescence of phalloidin was frequently increased at cell surface areas where the NK cell membrane interacted with the fungal hyphae (marked by arrow), concluding that actin polymerization is induced by fungal hyphae (Figure 7B). Interestingly, NK cells frequently attached in close proximity to the fungal septae (Supplementary Figure 4).

**Figure 8:**
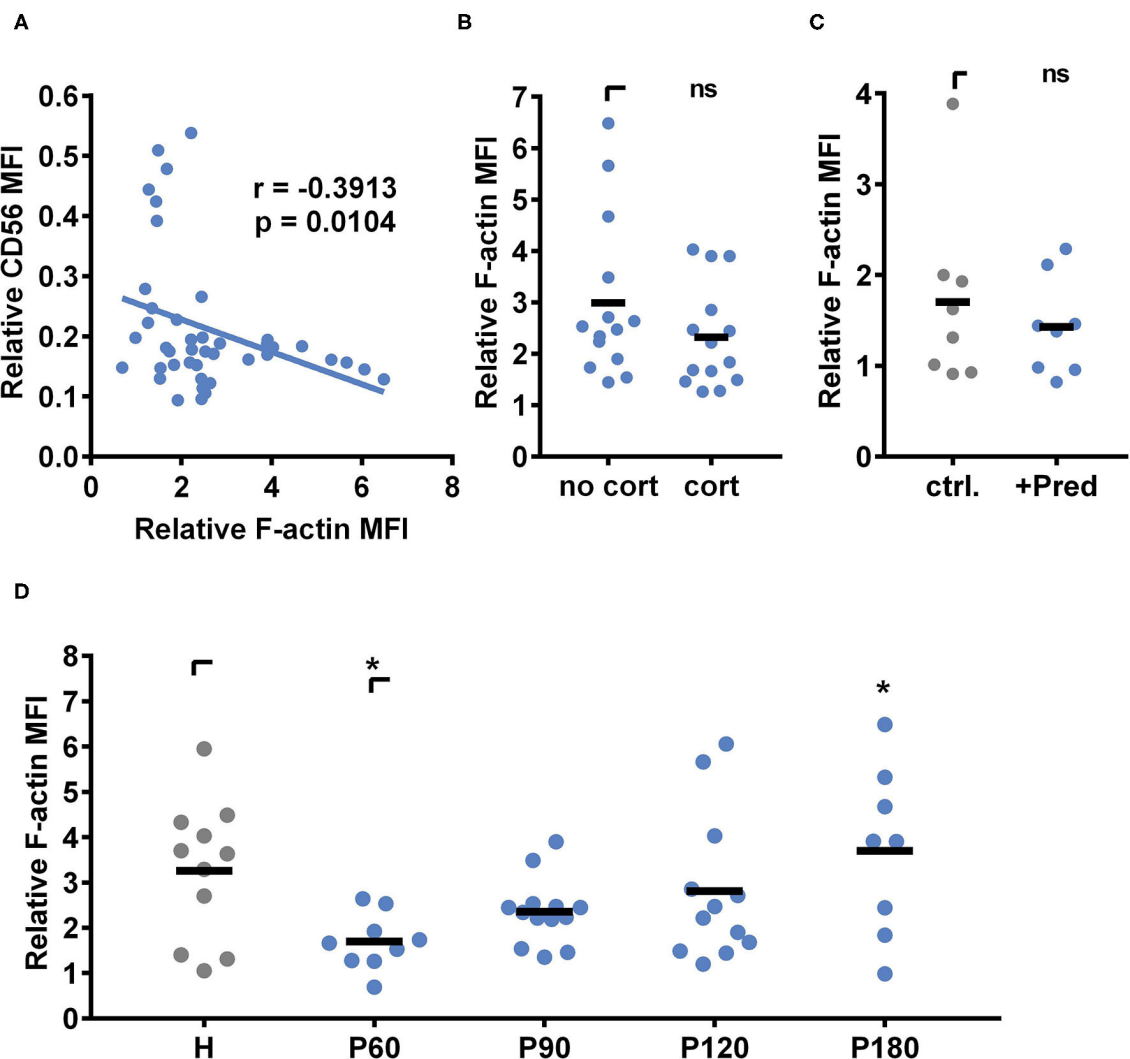
Actin induction following fungal stimulation recovers within 6 months after alloSCT. **(A,B,D)** NK cells were isolated from patients 60, 90, 120, and 180 days after alloSCT (P) or healthy controls (H). **(C)** NK cells were isolated from healthy controls and stimulated with 25 μg/ml prednisolone *ex vivo* for 40 h. **(A–D)** NK cells were cultured alone or with *A. fumigatus* germ tubes (MOI 0.5) for 6 h. Cells were stained for surface markers, treated with the F-Actin binding probe Sir647 for 50 min, and were analyzed by flow cytometry. Relative actin induction was calculated by the division of Sir647 MFI after fungal co-culture with Sir647 MFI of control cells. Data were acquired from **(A)** *n* = 44, **(B)** *n* = 14, **(C)** *n* = 8, and **(D)** *n* = 11 (H); *n* = 9 (P60), *n* = 13 (P90); *n* = 12 (P120); *n* = 8 (P180) different experiments. Data are displayed as **(B–D)** means. Significant differences were calculated by **(A)** two-tailed Spearman correlation, **(B)** unpaired *t*-test with Welch's correction**, (C)** Wilcoxon test, and **(D)** One-way ANOVA with FDR correction (F[4,48] = 3.112), and. Statistical significance is marked by an asterisk (*p < 0.05).

Next, we analyzed the actin cytoskeleton with live-cell staining using flow cytometry. NK cells obtained from healthy individuals or patients 60, 90, 120, or 180 days post alloSCT were incubated with *A. fumigatus* germ tubes for 6 h, co-cultures were harvested, and cells were incubated in 1 μM SiR 647 F-actin binding peptides (Figure 8). The induction of actin was calculated by dividing the actin signal after fungal co-culture by the actin signal before fungal co-culture (Figure 8). To analyze the dependency of CD56 binding on the actin induction, we plotted relative CD56 values obtained from NK cells after alloSCT against the individual fungal mediated actin induction (Figure 8A). Indeed, relative CD56 values negatively correlated with the induction of actin (*p* = 0.0104, two-tailed Spearman correlation), indicating that CD56 relocalization is dependent on fungal mediated actin polymerization (Figure 8A).

Since CD56 binding to the fungus was dependent on fungal mediated actin polymerization (Figure 8A) and CD56 binding was negatively affected by corticosteroid treatment (Figure 3B), we next analyzed whether actin polymerization changed during corticosteroid treatment. Therefore, we grouped patients into corticosteroid receiving and non-corticosteroid receiving cohorts and only included samples matching the time after alloSCT and further drug treatment. There were no significant differences in the fungal mediated actin induction observable between corticosteroid-receiving and non-corticosteroid receiving patients (Figure 8B). Also, *ex vivo* prednisolone treatment of healthy NK cells was not influencing fungal mediated actin induction (Figure 8C).

However, fungal mediated actin polymerization was dependent on the time point of blood collection after alloSCT. Interestingly, NK cells collected at early time points after alloSCT showed a weaker fungal mediated actin polymerization (mean at day 60: 1.69) in comparison to later time points (mean at day 180: 3.69) or healthy controls (mean: 3.26) (Figure 8D). Notably, CD16^+^ and CD16^−^ NK cells showed no differences in actin polymerization after *A. fumigatus* co-culture (Supplementary Figure 5), concluding that the differences between healthy individuals and patients after alloSCT are not the cause of different NK cell subset contributions.

**Figure 9:**
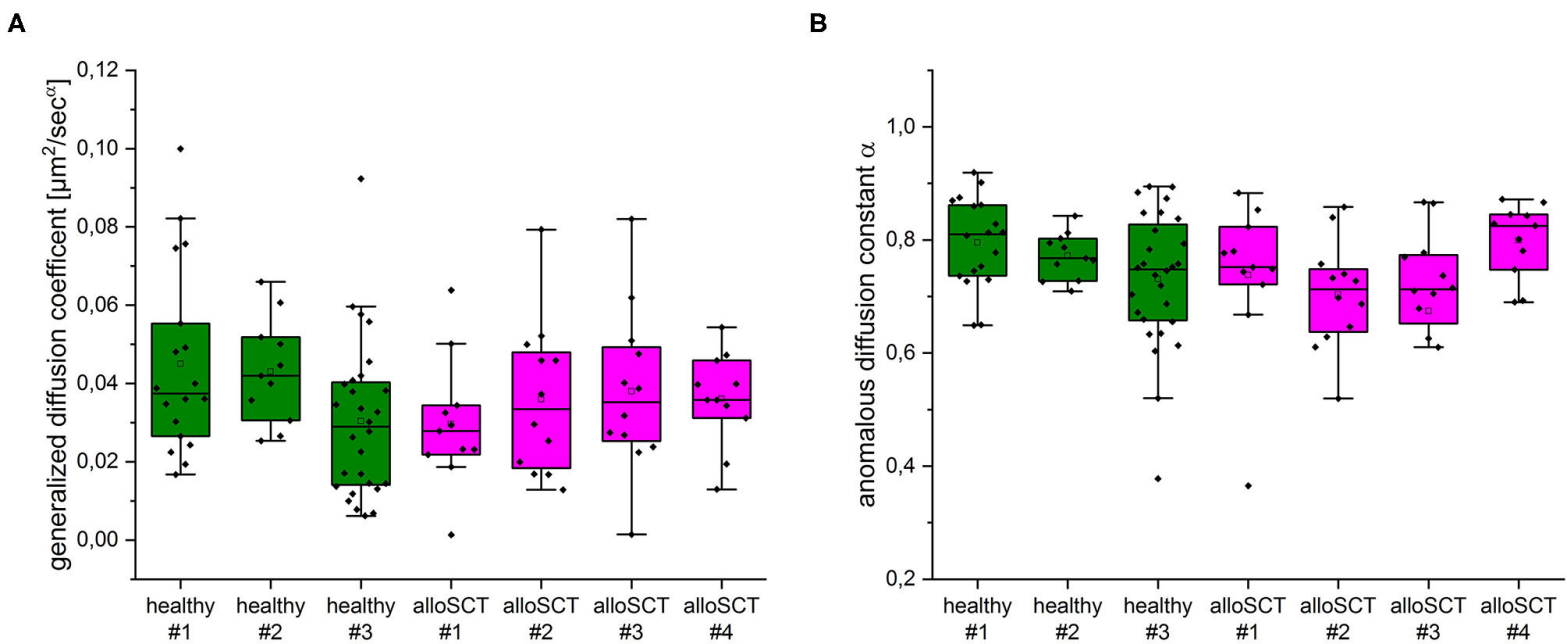
Single-molecule tracking of CD56 on primary NK cells of healthy and alloSCT patients isolated 120 days after alloSCT. **(A)** No significant differences in the ensemble diffusion coefficients derived from MSD analysis of individual trajectories between healthy (green) and alloSCT (magenta) NK cells. Median diffusion coefficients were between 0.028 and 0.042 μm^2^/s^α^. **(B)** Analysis of anomalous diffusion indicates subdiffusive behavior of CD56 on healthy (green) and alloSCT (magenta) NK cells with no significant differences between healthy and alloSCT trajectories. We analyzed at least 11 NK cells with numerous trajectories for each condition (healthy #1 *n* = 18, healthy #2 *n* = 11, healthy #3 *n* = 28, alloSCT #1 *n* = 11, alloSCT #2 *n* = 12, alloSCT #3 *n* = 12, alloSCT #4 *n* = 11).

To test, whether the impaired fungal binding in alloSCT derived NK cells might be due to changes in CD56 mobility we performed 2D single-molecule tracking (SMT) experiments of CD56 (Supplementary Video 1) as described previously (24, 25). We could not observe significant differences in the diffusion coefficient (Figure 9A), anomalous diffusion constant (Figure 9B) and bound fraction (data not shown) (38) between control and alloSCT NK cells. All tested parameters were quite robust and are in good agreement with previously published data (25). In order to follow single CD56 molecules simultaneously with the underlying actin cytoskeleton we performed SMT experiments in the presence of the fluorogenic far-red F-actin probe SiR700. Since fluorescence emission of labeled CD56 receptors and F-actin was detected by one camera and same filter settings, we had to bleach the strong F-actin signal before both signals had similar intensity regimes. CD56 molecules moved along individual actin filaments in the cell periphery (Supplementary Video 2) which is in good agreement with partial colocalization of CD56 and actin observed by SIM (**data not shown**).

## Discussion

Patients after alloSCT are at an increased risk of IA due to a long-term state of T/B cell deficiency (1). Besides, a lack of innate immune cells such as neutrophils further negatively impacts on the outcome of IA (39). Stuehler et al. correlated low NK cell counts and NK cell reconstitution with a higher risk of developing IA, which underlined the role of NK cells in the defense against *A. fumigatus* (19). The interaction of NK cells with *A. fumigatus* is mediated by the neuronal cell adhesion molecule (NCAM-1, CD56), which binds to interacting hyphae in an actin- and time-dependent manner. Blocking of CD56 was shown to reduce fungal mediated NK cell activation and the secretion of the immune-recruiting chemokines MIP-1α, MIP-1β, and RANTES (21).

In a recent publication from Santiago et al., the authors discuss NK cell exhaustion after overnight co-incubation of primary human NK cells with *A. fumigatus* (40). In this study, NK cell surface markers were down-regulated, possibly due to NK cell exhaustion, as described previously (41, 42). Since such extended NK cell-*A. fumigatus* co-cultivation might induce induction of NK cell apoptosis (21), we limited the co-cultivation of NK cells with *A. fumigatus* to a maximum of 6 h.

By comparing CD56 and CD16 expression levels on NK cells obtained after alloSCT and healthy controls, we confirmed the presence of more immature CD16^−^CD56^bright^ NK cells in the peripheral blood of allograft recipients which persisted in most of the patients until 180 days post-transplant. Nevertheless, we detected no significant differences in CD56 dependent fungal binding between NK cells derived from healthy individuals or alloSCT patients independent from the time point of blood draw. However, CD56 binding after alloSCT was not homogeneously distributed and showed outliers.

Considering the possibility that different drug treatments of alloSCT recipients might influence NK cell binding to fungal pathogens, we found CD56 binding to be inhibited in blood samples from patients receiving corticosteroid therapy. This therapy is mainly applied to treat acute or chronic-graft-vs.-host disease (GvHD). Interestingly, patients developing acute or chronic GvHD after alloSCT also have an increased risk of IA, especially when receiving corticosteroids (43). Corticosteroids have anti-inflammatory and immunosuppressive effects and are administered to prevent graft rejection after alloSCT (44). Additionally, several cell functions, e.g., cytotoxicity, cell metabolism, and cytokine production, are suppressed by glucocorticoid treatment (45–47).

Indeed, corticosteroid treatment inhibited the fungal mediated secretion of MIP-1α, MIP-1β, and RANTES in NK cells obtained at day 60–120 post-alloSCT. After 180 days, chemokine levels from corticosteroid treated NK cells normalized to that of healthy control levels which may be due to tapering of corticosteroid treatment over time, as corticosteroids are primarily used to treat GvHD after alloSCT (44, 48). Therefore, low-level doses of corticosteroids to later time points may only marginally impact chemokine secretion. The primary corticosteroid agent for treatment of a GvHD is prednisolone, thus we tested the effect of prednisolone on NK cells from healthy individuals *ex vivo*. Prednisolone abrogated the fungal mediated secretion of MIP-1α, MIP-1β, and RANTES in healthy NK cells, confirming detrimental effects on chemokine secretion and immune cell function.

Glucocorticoids intracellularly bind to glucocorticoid receptors which are then transported into the nucleus. GR can either bind to glucocorticoid response elements (GREs), and function as transcriptional inducers or repressors, or interact with other transcription factors, thereby influencing their target gene expression (49–52). In particular, glucocorticoids can inhibit NF-κB target gene expression by increasing the export rate of the activated p65 (RelA) NF-κB subunit from the nucleus to the cytoplasm (52). MIP-1α, MIP-1β, and RANTES are NF-κB target genes, and their biological function is to recruit leukocytes to sites of inflammation and the initiation of a protective Th1 response (53–57). Since NK cells secrete MIP-1α, MIP-1β, and RANTES after fungal binding by CD56, we hypothesize that CD56 might activate the NF-κB pathway after relocalization to the fungal interaction site.

Previous studies report that CD56 relocalization to the fungal interface is abolished after disruption of actin dynamics by cytochalasin D, concluding actin-dependent CD56 relocalization (21). We supported these findings by showing that a higher fungal mediated actin induction correlated with higher CD56 relocalization. We further demonstrated that the overall potential for fungal mediated actin polymerization was reduced in NK cells obtained after alloSCT. Interestingly, this was a time-dependent effect and actin defects recovered within 180 days post-alloSCT at which time actin induction was similar to healthy controls. Until now, it has not been clear why NK cells obtained early after alloSCT show a lower potential for actin polymerization. Actin polymerization is crucial for NK cell differentiation, activation, and cytotoxicity (58–60). Interestingly, the recent work from Lee and Mace has revealed that NK cell motility increases with cell maturation, concluding that the actin cytoskeleton may fully develop over time (59). Since actin dynamics are crucial for several cellular processes, this may impact further functions in NK cells and other cell types post-alloSCT that have to be investigated in future studies. Although CD56 relocalization to the hyphae is dependent on actin dynamics, the overall mobility and diffusion properties of this adhesion receptor in the plasma membrane is not influenced in alloSCT patients even upon corticosteroid treatment 120 days post-alloSCT. Thus, impaired CD56 function during corticosteroid therapy is probably not caused by defects in receptor mobility but rather by other mechanisms, e.g., hindered downstream signaling cascades. By using SMT we observed a small subset of CD56 molecules but did not account for the many CD56 isoforms and their degree of sialylation. Future studies should therefore unravel the role of CD56 isoforms as well as the time course after alloSCT on CD56 diffusion. Interestingly, CD56 mobility was very robust in all tested conditions which might reflect its important function in NK cell physiology.

We demonstrated that CD56 binding and the secretion of chemokines are impaired after corticosteroid treatment, suggesting that CD56 may activate NF-κB signaling after fungal binding. Since it was shown that NK cells have a protective effect on the outcome of IA (19), our data demonstrated that corticosteroid treatment might favor the development of IA also by suppressing NK cell function in addition to effects on other immune cells.

## Data Availability Statement

The raw data supporting the conclusions of this article will be made available by the authors, without undue reservation.

## Ethics Statement

The studies involving human participants were reviewed and approved by ethics committee of the University of Wuerzburg. The patients/participants provided their written informed consent to participate in this study.

## Author Contributions

EW and JS developed concepts, performed experiments, performed data analyses, and wrote the manuscript. UT developed concepts and wrote the manuscript. MW performed data analyses and wrote the manuscript. A-LS, KH, and LM performed experiments. JB and FG recruited blood donors and analyzed clinical data. CL analyzed clinical data. OK, MS, JLi, and CM provided discussion and contributed to the manuscript. HE and JLo developed concepts, supervised the study, and wrote the manuscript. All authors contributed to the article and approved the submitted version.

## Conflict of Interest

The authors declare that the research was conducted in the absence of any commercial or financial relationships that could be construed as a potential conflict of interest.
