# Supplementary material for: Reconstituting NK Cells After Allogeneic Stem Cell Transplantation Show Impaired Response to the Fungal Pathogen Aspergillus fumigatus
Source: Front Immunol. 2020 Sep 10;11:2117. doi: 10.3389/fimmu.2020.02117 (PMC7511764; doi:10.3389/fimmu.2020.02117)
Supplement: Supplementary file 3 [file Data_Sheet_1.PDF]

Supplementary Information

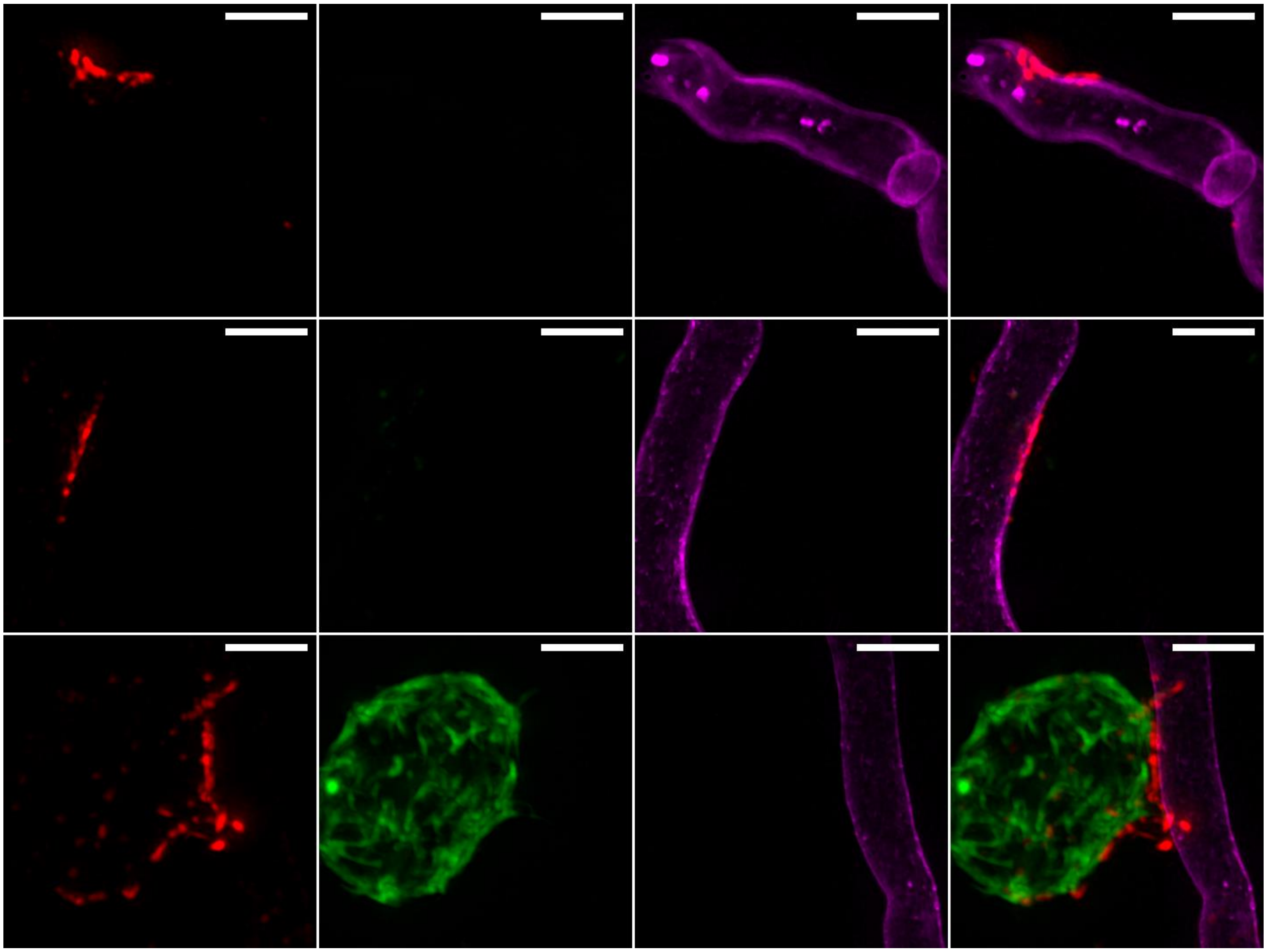

Supplementary Figure 1: The remaining CD56-positive interaction site on the fungal surface after physical separation of co-cultures. First two rows show example images from NK cell CD56 contact sites (red) after physical separation of NK cells (green) from *A. fumigatus* (magenta). Remaining CD56 signal at the hyphae indicates a strong molecular interaction, whereas whole NK cells (green) were washed away. NK cells were labelled with phalloidin and *A. fumigatus* with calcofluor. Scale bar, 3 µm.

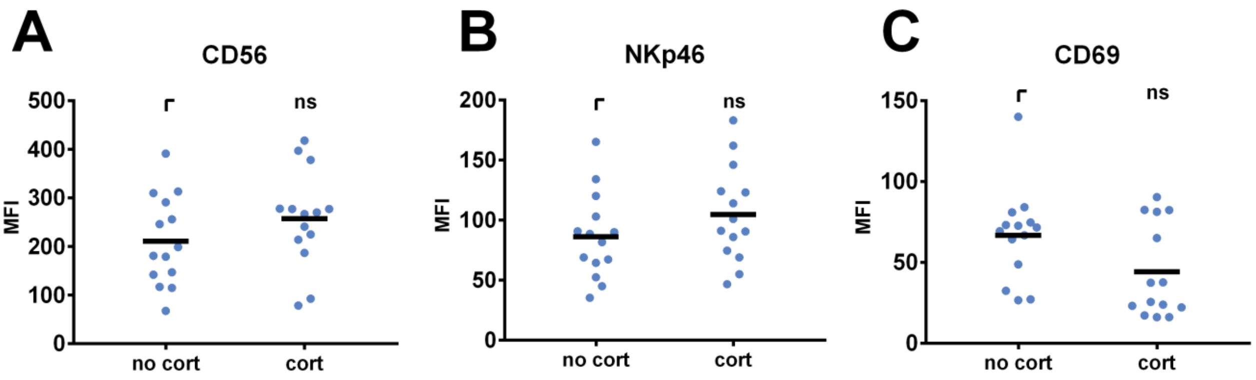

Supplementary Figure 2: Surface expression of CD56, NKp46, and CD69 on NK cells obtained from corticosteroid recipients. NK cells derived after alloSCT were incubated with 1000 U/ml IL-2 overnight before the medium was exchanged, and NK cells were cultured for 6 h in RPMI + FCS. The surface expression of **(A)** CD56, **(B)** NKp46, and **(C)** CD69 was determined by flow cytometry. Mean fluorescence intensities (MFI) were determined in NK cells obtained from patients with (cort) or without corticosteroid (no cort) treatment. Samples were matched regarding time after alloSCT and further drug treatment. Data were acquired from n = 14 experiments. Data are displayed as means. Statistical analysis was performed by unpaired t-test with Welch’s correction.

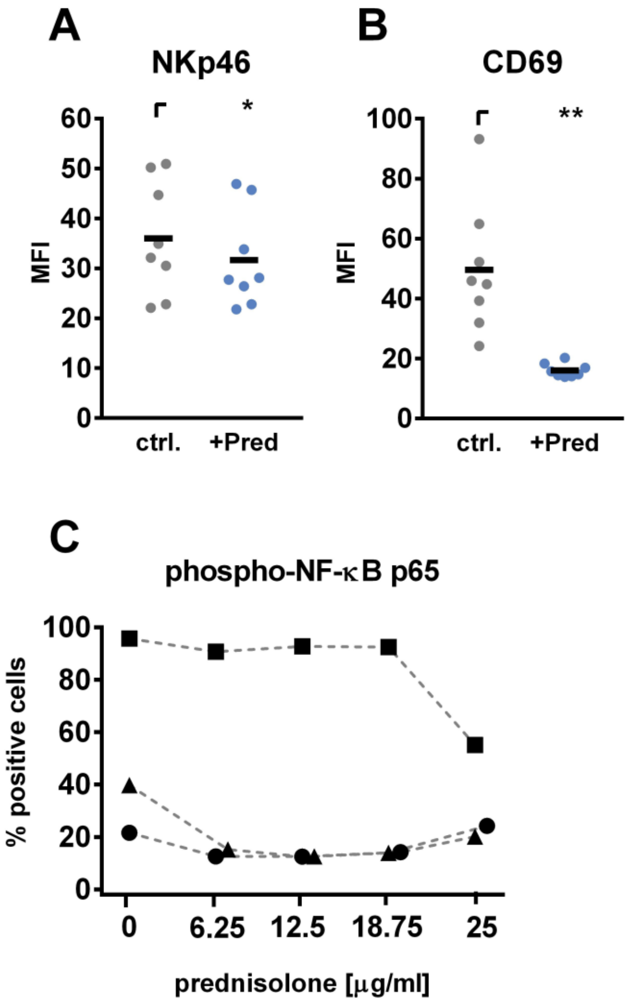

Supplementary Figure 3: Prednisolone treatment of healthy NK cells decreases the expression of the activation markers NKp46 and CD69 and intracellular phosphor-NF-κB p65 peptide. NK cells were treated with 25 µg/ml prednisolone in the presence of 1000 U/ml IL-2 for 40 h. The medium was exchanged, and NK cells were either cultured alone or with *A. fumigatus* germ tubes (MOI 0.5) for 6 h. Cells were analyzed regarding the surface expression of **(A)** NKp46 and **(B)** CD69 using flow cytometry. Data are displayed as means. Statistical analyses were performed by paired t-test and significant differences are marked by asterisks (* $p < 0.05$, ** $p < 0.01$). **(C)** NK cells were treated with increasing concentrations of prednisolone (0, 6.25, 12.5, 18.75, and 25 µg/ml) in the presence of 1000 U/ml IL-2 for 40 h. NK cells were stained with surface antibodies (anti-CD3), and were intracellularly stained with anti-NF-κB p65 (pS529) antibody using the BD Cytofix/Cytoperm™ protocol. Data were acquired from **(A, B)** n = 8, **(C)** n = 3 different experiments.

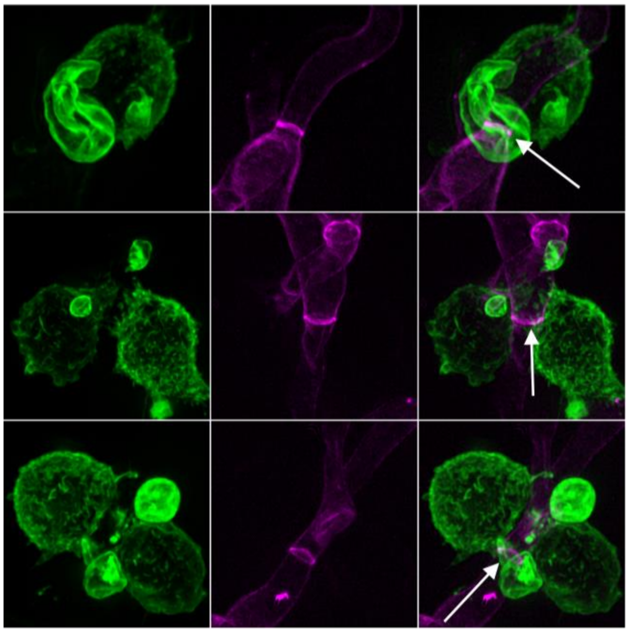

Supplementary Figure 4: NK cells interact at *A. fumigatus* septae. NK cells were labelled with phalloidin (green) and *A. fumigatus* with calcofluor (magenta). Frequently, NK cells adhered at fungal septae (white arrow) and showed strong actin induction.

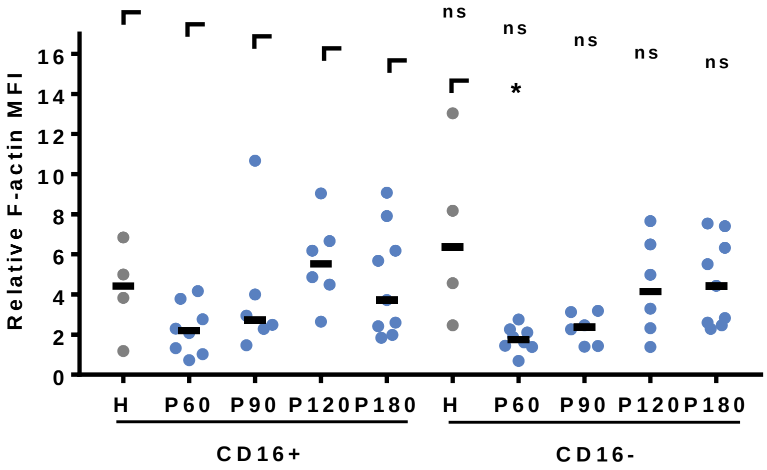

Supplementary Figure 5: NK cells were isolated from patients 60, 90, 120, and 180 days after alloSCT (P) or healthy controls (H). For analysis, NK cells were pre-stimulated with 1000 U/ml IL-2 overnight and afterward co-cultured with *A. fumigatus* germ tubes (MOI 0.5) or alone for 6 h. NK cells were treated with the F-Actin binding probe Sir647 for 50 min before cells were analyzed by flow cytometry. Relative actin induction was calculated by the division of Sir647 MFI after fungal co-culture with Sir647 MFI of control cells. Data were acquired from n = 4 (H); n = 8 (P60); n = 6 (P90); n = 6 (P120); n = 9 (P180) different experiments. Data are displayed as medians. Significant differences were calculated by Kruskal-Wallis test with FDR correction to compare within NK cell subsets, and Wilcoxon test to compare between NK cell subsets. Statistical significance is marked by an asterisk (* $p < 0.05$).

Supplementary Video 1: Single-molecule tracking of CD56 on human NK cells. NK cells were isolated from patients 120 days after alloSCT or healthy individuals and labelled with anti-CD56 antibodies as described. Example video from one experiment showing CD56 receptors moving on the basal plasma membrane. Single spots are marked by magenta circles and local tracks are shown as yellow lines. The video was generated with the help of the Fiji plugin TrackMate (Tinevez et al., 2017, Schindelin et al., 2012). Scale bar, 2 µm.

Supplementary Video 2: Single-molecule tracking of CD56 on human NK cells in combination with actin visualization. NK cells were isolated from patients 120 days after alloSCT or healthy individuals and labelled with anti-CD56 antibodies and SiR700 actin as described. Example video from one experiment showing bright CD56 receptors moving along individual actin filaments in the NK cell periphery.

References

Tinevez, Perry, Schindelin, Hoopes, Reynolds, Laplantine, . . . Eliceiri, TrackMate: An open and extensible platform for single-particle tracking. *Methods* 115, 80-90 (2017).

Schindelin, Arganda-Carreras, Frise, Kaynig, Longair, Pietzsch, . . . Cardona, Fiji: an open-source platform for biological-image analysis. *Nat Methods* 9, 676-682 (2012).
